# Chronic sialadenitis caused by sarcoidosis: A Case Report

**DOI:** 10.1590/S1808-86942010000100022

**Published:** 2015-10-17

**Authors:** Jose Castro Lima Geraldes Filho, Helenimarie Schaer Barbosa, Delano O. Souza, Alisio A. Prates

**Affiliations:** 1 ENT Specialist - Hosp. Santa Casa/SSA-BA. MSc in Head and Neck Surgery - Hospital Heliópolis-SP, Assistant Physician - Head and Neck Department - Hospital Aristides Maltez-SSA/BA; 2 Adjunct Professor - Federal University of Bahia Medical School - UFBA, Assistant Physician - Pathology Department - Hospital Aristides Maltez SSA/BA; 3 Resident physician in traumatology and maxillofacial surgery - Hospital Santo Antonio-SSA/BA; 4 Dentistry student - UFBA. Serviço de Cirurgia de Cabeça e Pescoço do Hospital Aristides Maltez

**Keywords:** submandibular gland, granulomatous, sarcoidosis

## INTRODUCTION

Sarcoidosis is a systemic granulomatous, non-infectious multifocal disorder of unknown etiology, characterized by the presence of non-caseous epithelioid granuloma in the tissue^1^,^2^. About 20 to 40% of the patients are symptom-free and their disease is found through routine radiographic tests^1^. The hypothetical causal agents include environmental and autoantigen aspects^1^,^3^. The goal is to report a rare case^4^ of chronic sialoadenitis having sarcoidosis as the cause.

## CASE REPORT

Male patient, 41 years old, had a face swelling, reduced salivation, red eyes and dysphagia for months. He had a uniform and bilateral enlargement of the parotid and submandibular gland (Fig. B) - painless, firm, smooth and mobile; with hyperemia of the ocular conjunctiva (Fig. A). During routine exams, the chest x-ray showed an image suggesting the presence of a peribronchovascular infiltrate, without clinical significance. The patient was referred to incisional biopsy of the right submandibular gland and smaller salivary gland of the lower lip. Histopathology reported Chronic Granulomatous Sialoadenitis, a matching diagnosis of sarcoidosis. The patient returned to the ward with a history of past use of non-hormonal anti-inflammatory agent (without medical instruction), with total remission of signs and symptoms.

**Figure 1:**
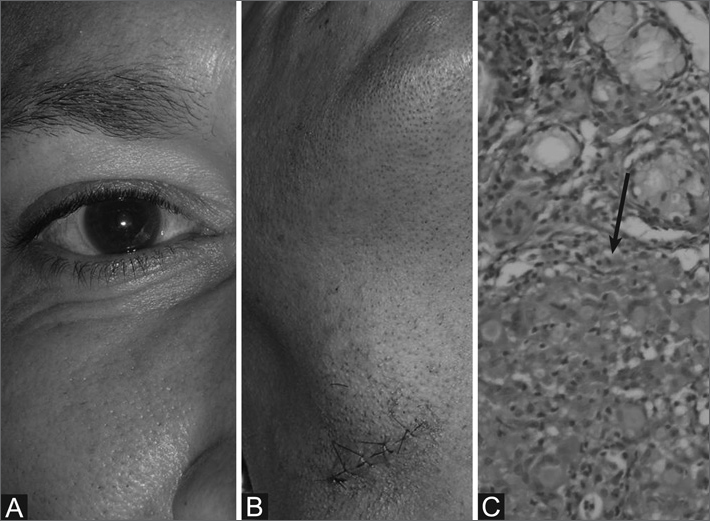
Sarcoidosis - Figure A- conjunctiva hyperemia; Figure B - biopsied mandibular hypertrophy; Figure C - salivary gland with granulomas made up of epithelioid cells and multinucleated gigantic cells (arrow).

## DISCUSSION

Sarcoidosis is usually acute in onset, with more common clinical symptoms of dyspnea, dry cough, chest pain, fever, malaise, fatigue, arthralgia and weight loss1 and, about 20% of the patients are assymptomatic1. The lungs, lymph nodes, skin, eyes and salivary glands are the most affected organs^1^,^2^. Skin lesions such as plaques and nodules, nasal mucosa and liver granulomas may also be present^2^.

The lymphoid tissue is affected in almost all the cases and the salivary gland swelling, xerostomia and eye involvement may combine and mirror Sjögren Syndrome^6^, which led us to do the lower lip salivary gland biopsy.

The respiratory system is usually the one most involved, and 90% of the patients have an abnormal chest x-ray^1,^^5,^^3^. The most common symptoms are dyspnea, fever, fatigue and dry cough^1^,^2^, which did not happen to our patient.

Eye lesions are seen in approximately 25%^1^. Inflammation on the anterior uveal interval is the most common eye lesion. The patients complain of visual alteration and photophobia. The lesions are chronic and can progress to blindness^1^,^2^.

It rarely involves the oral cavity, more frequently appearing as an isolate submucosal mass or area of granularity^1^,^6^. The facial is the most affected cranial nerve and it can also involve the inferior alveolar nerve^1^. Our patient complained of dysphagia; however, we believe it was a symptom related to reduced salivation.

Histopathology helps in the diagnosis, which was fundamental in our case. There were no morphological aspects suggesting Miculicz/Sjögren syndrome diseases and both fungi (Grocott) and mycobacterium (Ziehl-Neelsen) tests turned out negative. In figure C we notice granulomas made up of epithelioid and gigantic multinucleated cells.

Diagnosis is based on clinical history, radiographic characteristics, histological evidence and ruling out other diseases capable of producing similar clinical or histological manifestations1. Some laboratorial abnormalities such as hypercalcemia and hypercalcinuria may be present. The serum levels of the Angiogenesis Converting Enzyme may be elevated in 60% and, are useful in monitoring disease development and remission. A skin test for sarcoidosis, the Kveim test can be performed^1^,^2^; however, it is rarely used.

A 3 to 12 months observation period is usually recommended to establish disease course1,6. Treatment is usually indicated for severe eye involvement; symptomatic lungs; neurological, renal, cardiac and skin manifestations which affect the face and cause spleen enlargement^1^,^6^. The treatment of choice for sarcoidosis is steroids.

We believe that the use of non-hormonal anti-inflammatory agents by our patient for less than ten days are not the cause for disease remission, but rather a spontaneous resolution.

## FINAL REMARKS

Histopathological test, radiographic tests and clinical manifestations are fundamental for the diagnosis. Treatment is based on steroids or observation only.
